# Evolution of the F_0_F_1_ ATP Synthase Complex in Light of the Patchy Distribution of Different Bioenergetic Pathways across Prokaryotes

**DOI:** 10.1371/journal.pcbi.1003821

**Published:** 2014-09-04

**Authors:** Vassiliki Lila Koumandou, Sophia Kossida

**Affiliations:** Bioinformatics & Medical Informatics Team, Biomedical Research Foundation, Academy of Athens, Athens, Greece; Hellas, Greece

## Abstract

Bacteria and archaea are characterized by an amazing metabolic diversity, which allows them to persist in diverse and often extreme habitats. Apart from oxygenic photosynthesis and oxidative phosphorylation, well-studied processes from chloroplasts and mitochondria of plants and animals, prokaryotes utilize various chemo- or lithotrophic modes, such as anoxygenic photosynthesis, iron oxidation and reduction, sulfate reduction, and methanogenesis. Most bioenergetic pathways have a similar general structure, with an electron transport chain composed of protein complexes acting as electron donors and acceptors, as well as a central cytochrome complex, mobile electron carriers, and an ATP synthase. While each pathway has been studied in considerable detail in isolation, not much is known about their relative evolutionary relationships. Wanting to address how this metabolic diversity evolved, we mapped the distribution of nine bioenergetic modes on a phylogenetic tree based on 16S rRNA sequences from 272 species representing the full diversity of prokaryotic lineages. This highlights the patchy distribution of many pathways across different lineages, and suggests either up to 26 independent origins or 17 horizontal gene transfer events. Next, we used comparative genomics and phylogenetic analysis of all subunits of the F_0_F_1_ ATP synthase, common to most bacterial lineages regardless of their bioenergetic mode. Our results indicate an ancient origin of this protein complex, and no clustering based on bioenergetic mode, which suggests that no special modifications are needed for the ATP synthase to work with different electron transport chains. Moreover, examination of the ATP synthase genetic locus indicates various gene rearrangements in the different bacterial lineages, ancient duplications of *atpI* and of the beta subunit of the F_0_ subcomplex, as well as more recent stochastic lineage-specific and species-specific duplications of all subunits. We discuss the implications of the overall pattern of conservation and flexibility of the F_0_F_1_ ATP synthase genetic locus.

## Author Summary

Bacteria and archaea are the most primitive forms of life on Earth, invisible to the naked eye and not extremely varied or impressive in their appearance. Nevertheless, they are characterized by an amazing metabolic diversity, especially in the different processes they use to generate energy in the form of ATP. This allows them to persist in diverse and often extreme habitats. Wanting to address how this metabolic diversity evolved, we mapped the distribution of nine bioenergetic modes across all the major lineages of bacteria and archaea. We find a patchy distribution of the different pathways, which suggests either frequent innovations, or gene transfer between unrelated species. We also examined the F-type ATP synthase, a protein complex which is central to all bioenergetic processes, and common to most types of bacteria regardless of how they harness energy from their environment. Our results indicate an ancient origin for this protein complex, and suggest that different species, without necessitating major innovation, used their pre-existing ATP synthase and adapted it to work with different bioenergetic pathways. We also describe gene duplications and rearrangements of the ATP synthase subunits in different lineages, which suggest further flexibility and robustness in the control of ATP synthesis.

## Introduction

Bacteria and archaea use diverse bioenergetic electron transport chains to generate ATP. Apart from photosynthesis and aerobic respiration, many other bacterial and archaeal bioenergetic pathways have been characterized in considerable biochemical detail (e.g. [1], [2], [3], [4], [5], [6], [7], [8], [9], [10], [11], [12]). However, the origins of the diversity of bioenergetic pathways, and their evolutionary relationships, have so far received relatively little attention. Did each pathway evolve independently or did they all evolve from a common ancestral metabolic mode? As in organismal evolution, it is likely that there were some novel innovations and that parts of pre-existing pathways were co-opted to evolve into new pathways. Molecular evolutionary studies of shared proteins amongst prokaryotes, coupled to data from the geological record, indicate that the vast majority of extant bioenergetic pathways evolved within the first billion years from the origin of life on earth [13], [14] and have since been mostly characterized by stasis [15]. Interestingly, when 16S rRNA phylogenetic analysis is carried out for a variety of prokaryotes, organisms that utilize different bioenergetic pathways don't group into clear monophyletic groups, i.e. closely related organisms can utilize quite distinct bioenergetic strategies [16], [17]. This may be due to horizontal gene transfer [18], and highlights the challenge of deciphering the evolution of these pathways.

While most previous studies have focused on comparison of the organisms that harbour the bioenergetic machinery, direct comparisons of the proteins that compose the bioenergetic machinery has been more limited. Most bioenergetic pathways use an electron transport chain (ETC) to generate a proton gradient across the membrane, and the energy released by the flow of electrons to compensate for this gradient is then used by the ATP synthase to generate ATP. The electron transport chains of disparate pathways have a similar general structure, being composed of protein complexes acting as electron donors and acceptors, with a central cytochrome *bc*-type complex and mobile electron carriers between them. Three scenarios are envisaged for the early evolution of energetic flexibility in the bacteria and the archaea: (i) each bioenergetic pathway evolved independently, (ii) all bioenergetic pathways evolved from a “simpler” ancestral metabolism, (iii) some new metabolic capabilities evolved by the modification of pre-existing pathways. The third scenario is the most likely, and has been highlighted through detailed analysis of the bioenergetic protein complexes, e.g. for oxygenic and anoxygenic photosynthesis [19], [20], [21].

The unprecedented availability of genomic data enables us to address evolutionary questions relating to the events that led to the emergence of this metabolic diversity early in the evolution of life on Earth. Although various studies have looked at the evolution of ATP synthases across the bacteria and the archaea (e.g. [22], [23], [24]), these have mostly addressed the relative relationships between the F-V- and A-type ATPases, and no study has looked at organisms spanning the full bioenergetic diversity of bacteria. We chose to examine the F_0_F_1_ ATP synthase complex, common to nine bioenergetic modes, and sampled a large variety of species across all major lineages to establish their homology and evolutionary relationships. We first asked whether the evolution of the ATP synthase complexes in these species agrees with the 16S rRNA phylogeny, i.e. whether they cluster according to the type of ETC, or based on taxonomic groups. This enables us to check for horizontal gene transfer events concerning the ATP synthase, as well as for putative specific modifications in the ATP synthase subunits associated with each bioenergetic mode. We also examined the structure of the F_0_F_1_ ATP synthase genetic locus, and report a variety of both ancient and recent gene duplications and rearrangements.

## Results

### No monophyly of bioenergetic modes

In this study, we focused on nine pathways most of which have been well characterized at the biochemical level, and for which enough sequence information is available to enable assessment of the diversity within each group as well as inter-group relationships:

- Oxygenic photosynthesis (cyanobacteria, e.g. *Synechococcus*)
- Anoxygenic photosynthesis (green sulfur bacteria, e.g. *Chlorobium*; green non-sulfur bacteria, e.g. *Chloroflexus*; proteobacteria, e.g. *Chromatium*, *Rhodospirillum*, *Rhodopseudomonas*; heliobacteria, e.g. *Heliobacterium*)
- Methanogenesis (methanogenic archaea, e.g. *Methanosarcina*, *Methanococcus*)
- Sulfate reduction (bacteria, e.g. *Desulfovibrio*, and archaea, e.g. *Archaeoglobus*)
- Sulfur reduction (bacteria, e.g. *Sulfurospirillum*, and archaea, e.g. *Ignicoccus*)
- Sulfur oxidation (e.g. *Sulfurimonas*)
- Iron oxidation (bacteria, e.g. *Acidithiobacillus*, and archaea, e.g. *Ferroplasma*)
- Iron reduction (e.g. *Geobacter*)
- Aerobic respiration (heterotrophs, e.g. *E. coli*)

**Figure 1:**
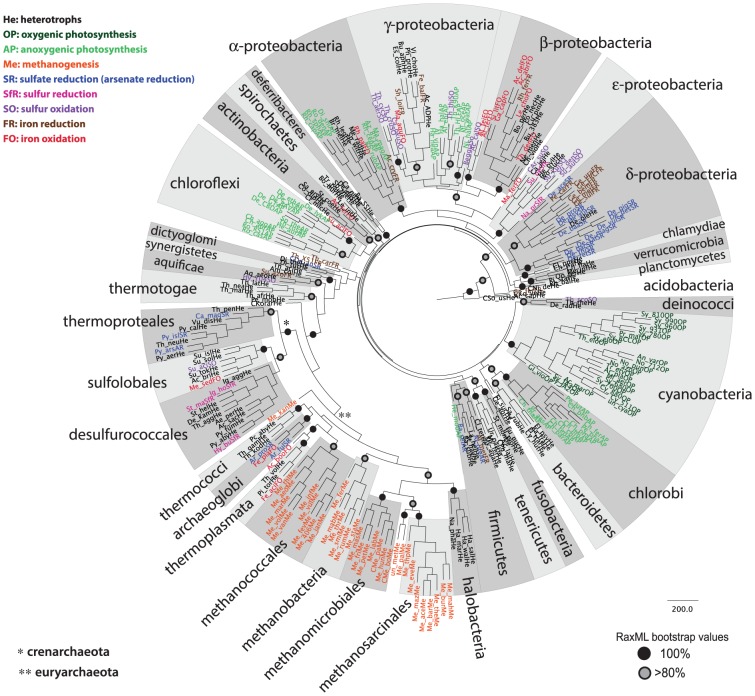
Phylogenetic reconstruction based on 16S rRNA sequences to map the taxonomic distribution of bioenergetic pathways. 272 prokaryotic species are shown, whose full genome sequence is available, and which represent the full diversity of bacteria and archaea, colour-coded based on their bioenergetic mode. Bootstrap values for highly supported nodes have been replaced by symbols, as indicated. The full species names, as well as details and accession numbers for all sequences used are given in Table S1. The tree shown was produced by RaxML, and its topology broadly agrees with the one produced by PhyML (the analysis based on MrBayes did not converge after 5 million generations when all sequences were included; however, when the bacteria and the archaea were examined separately, the MrBayes analysis also agreed with the RaxML and PhyML results).

Species, whose complete genomes are available, were chosen to represent all major lineages of bacteria and archaea, and all the above bioenergetic modes. Information about the metabolism (bioenergetic mode) of each species was collected from the species description at the NCBI BioProject database, as well as from the Integrated Microbial Genomes database. Full details of the 198 bacteria and 74 archaea species selected are given in Table S1, while the number of species from each lineage, and each bioenergetic mode is shown in Table 1. As has been observed in previous analyses [16], [17], [18], certain bioenergetic modes can be shared by quite distinct taxonomic groups. Indeed, as demonstrated by 16S rRNA phylogenetic analysis of the organisms examined here (Figure 1), species which utilize the same bioenergetic modes do not always segregate in monophyletic groups.

**Table 1:** The distribution of bioenergetic modes across taxonomic lineages suggests rampant horizontal gene transfer, or multiple independent origins.

		number of species per bioenergetic mode									Total # of species	Total # of bioenergetic modes	
	lineage^a^	He	OP	AP	Me	SR/AR	SfR	SO	FR	FO			
**bacteria**	acidobacteria	3	0	0	0	0	0	0	1	0	4	2	
	actinobacteria	4	0	0	0	0	0	0	0	1	5	2	
	aquificae	1	0	0	0	0	0	1	1	0	3	3	
	bacteroidetes	5	0	0	0	0	0	0	0	0	5	1	
	chlamydiae	2	0	0	0	0	0	0	0	0	2	1	
	chlorobi	0	0	11	0	0	0	0	0	0	11	1	
	chloroflexi	0	0	13	0	0	0	0	0	0	13	1	
	**chrysiogenetes (Ba_S5He)**	1	0	0	0	0	0	0	0	0	1	1	
	cyanobacteria	0	25	0	0	0	0	0	0	0	25	1	
	deferribacteres	2	0	0	0	0	0	0	0	0	2	1	
	deinococci	2	0	0	0	0	0	1	0	0	3	2	
	dictyoglomi	2	0	0	0	0	0	0	0	0	2	1	
	**fibrobacteres (Fi_sucHe)**	1	0	0	0	0	0	0	0	0	1	1	
	firmicutes	4	0	1	0	3	0	0	3	1	12	5	
	fusobacteria	3	0	0	0	0	0	0	0	0	3	1	
	**gemmatimonadetes (Ge_aurHe)**	1	0	0	0	0	0	0	0	0	1	1	
	**nitrospirae (CNi_deHe)**	1	0	0	0	0	0	0	0	0	1	1	
	planctomycetes	2	0	0	0	0	0	0	0	0	2	1	
	proteobacteria_alpha	4	0	8	0	0	0	0	1	1	14	4	
	proteobacteria_beta	6	0	0	0	0	0	0	1	6	13	3	
	proteobacteria_gamma	5	0	9	0	0	0	7	2	4	27	5	
	proteobacteria_delta	1	0	0	0	13	0	0	6	0	20	3	
	proteobacteria_epsilon	3	0	0	0	0	2	4	0	0	9	3	
	spirochaetes	4	0	0	0	0	0	0	0	0	4	1	
	synergistetes	2	0	0	0	0	0	0	0	0	2	1	
	tenericutes	5	0	0	0	0	0	0	0	0	5	1	
	thermotogae	5	0	0	0	0	0	0	0	0	5	1	
	verrucomicrobia	3	0	0	0	0	0	0	0	0	3	1	
**crenarchaea**	**acidilobales (Ac_sacHe)**	1	0	0	0	0	0	0	0	0	1	1	
	desulfurococcales	7	0	0	0	0	3	0	0	0	10	2	
	sulfolobales	4	0	0	0	0	0	1	0	1	6	3	
	thermoproteales	5	0	0	0	3	0	0	0	0	8	2	
**euryarchaea**	archaeglobi	0	0	0	0	2	0	0	0	1	3	2	
	halobacteria	4	0	0	0	0	0	0	0	0	4	1	
	methanobacteria	0	0	0	6	0	0	0	0	0	6	1	
	methanocellales	0	0	0	2	0	0	0	0	0	2	1	
	methanococcales	0	0	0	10	0	0	0	0	0	10	1	
	methanomicrobiales	0	0	0	7	0	0	0	0	0	7	1	
	**methanopyri (Me_kanMe)**	0	0	0	1	0	0	0	0	0	1	1	
	methanosarcinales	0	0	0	8	0	0	0	0	0	8	1	
	thermococci	3	0	0	0	0	0	0	0	0	3	1	
	thermoplasmata	2	0	0	0	0	0	0	0	1	3	2	
	**unclassified in NCBI (Ac_booFO)**	0	0	0	0	0	0	0	0	1	1	1	
korarchaea	**CKorarHe**	1	0	0	0	0	0	0	0	0	1	1	

		He	OP	AP	Me	SR/AR	SfR	SO	FR	FO			
	Total # of species	99	25	42	34	21	5	14	15	17	272		
	Total # of lineages	34	1	5	7	4	2	5	7	9	46		
	Common between bacteria & archaea	yes	no^b^	no^b^	no^c^	yes	Yes	yes	no^b^	yes			
	Inferred # of independent origins	1^d^	1	4^e^	1^f^	4	2	4^e^	4^e^	5^g^	Total inferred origins		26
	or… Inferred # of HGT events^h^	0^d^	0	3^e^	0^f^	3	1	3^e^	3^e^	4^g^	Total inferred HGT events		17

He: heterotrophs/respiration.

OP: oxygenic photosynthesis.

AP: anoxygenic photosynthesis.

Me: methanogenesis.

SR/AR: sulfate/arsenate reduction.

Sfr: sulfur reduction.

SO: sulfur oxidation.

FR: iron reduction.

FO: iron oxidation.

a = lineages represented by only one species are shown in bold and the species name abbreviation is given.

b = only found in bacteria.

c = only found in the archaea.

d = ancient origin assumed (patchy distribution likely due to secondary loss, but see [37]).

e = assuming a common origin for the subgroups of the proteobacteria.

f = ancient origin before common ancestor of the crenarchaea.

g = assuming a common origin for the archaeglobi, the thermoplasmata and Ac_booFO.

h = assuming one origin.

Inferring the origin of each bioenergetic mode is therefore confounded by their patchy distribution among the prokaryotes. Oxygenic photosynthesis is the only bioenergetic mode which is unique to a lineage (the cyanobacteria). Oxidative phosphorylation (respiration) is shared by the greatest variety of lineages, and as such, can be considered as an ancient mode of generating energy in both the bacteria and the archaea, while methanogenesis is found in seven lineages within the euryarchaea, and as such can be considered ancient to this group However, anoxygenic photosynthesis, sulfur reduction, sulfate reduction, sulfur oxidation, iron reduction and iron oxidation are found in more than one lineage, which are not closely related. The presence of the same pathway in these distinct lineages, can come about by one of three processes: either (a) all bioenergetic modes were found in the common ancestor of these lineages, and some have been lost from some lineages, or (b) bioenergetic modes were acquired by distinct lineages by horizontal gene transfer (HGT), or (c) some electron transport chains originated multiple times independently in different lineages. The most parsimonious explanation is probably HGT, since, based on the phylogenetic tree of Figure 1, and as summarized at the bottom of Table 1, the distribution of bioenergetic pathways can be explained by up to 26 independent origins or, alternatively, 17 horizontal gene transfer events. Four HGT events can be inferred for iron oxidation, three HGT events can be inferred for anoxygenic photosynthesis, sulfate reduction, sulfur oxidation and iron reduction, and one HGT event can explain the distribution of sulfur reduction (Table 1). These inferences are based on minimal assumptions of lineage groupings (e.g. for the alpha– beta- gamma- and delta-proteobacteria) as the branching order of prokaryotic lineages is still largely unresolved [25], [26], [27], [28], [29]; the lineage-groupings seen in a more recent and better-resolved bacterial phylogeny [30] still do not change these numbers. Moreover, while iron reduction, and anoxygenic photosynthesis are specific to the bacteria, the other modes (sulfate reduction, sulfur reduction, sulfur oxidation, and iron oxidation) are found in both bacteria and archaea. Notably, certain lineages seem more prone to bioenergetic diversity than others. For example, five bioenergetic modes are seen within the gamma-proteobacteria and the firmicutes; four bioenergetic modes are found within the alpha-proteobacteria, three bioenergetic modes are found within the beta- the delta- and the epsilon-proteobacteria, the aquificae, and the sulfolobales; two bioenergetic modes are found within the deinococci, the acidobacteria, the actinobacteria, the thermoproteales, the desulfurococcales, and the thermoplasmata, while sulfate reduction and iron oxidation are both seen in the archaeoglobi. However, this may be influenced by how many complete genomes are available per lineage, and how well this represents the true diversity in each lineage [31]. This picture may thus change in the future, as more diverse organisms are sequenced.

### Phylogenetic analysis of the ATP synthase genes

As the ATP synthase complex is common to all the electron transport chains of the studied bioenergetic modes, we chose to study the evolution of this complex in the different lineages. To examine whether the ATP synthase complex which is associated with the different bioenergetic modes was also subject to HGT, we performed phylogenetic analysis of all the protein subunits of the F_0_F_1_ ATP synthase, as this is shared by most of the bacterial lineages. However, archaea and certain bacterial species/lineages lack ATPF_0_F_1_ altogether, and have ATPV instead: *Clostridium tetani* and *Thermoanaerobacter* sp. X513 (clostridia), *Chlamydia trachomatis* and *Chlamydophila pneumoniae* (chlamydiae), *Deinococcus radiodurans*, *Thermus scotoductus* and *Thermus thermophilus* (deinococci), *Fibrobacter succinogenes* (fibrobacteres), *Borrelia burgdorferi*, *Spirochaeta thermophila* and *Treponema pallidum* (spirochaetaceae), *Aminobacterium colombiense* and *Thermanaerovibrio acidaminovorans* (synergistetes), *Candidatus Phytoplasma mali* (mollicutes). As most subunits of the V-type and the F-type ATPases are not homologous [24], we chose to focus solely on the F_0_F_1_ ATP synthase.

Gene sequences were identified using KEGG orthology annotations, both by searching the KEGG orthology tables, and by manual searches in IMG (for the species not included in KEGG). The bacterial F_0_F_1_ ATP synthase complex is composed of the F_0_ subcomplex, which is embedded in the membrane, and the F_1_ subcomplex which protrudes on the side of the membrane towards which the protons exit following the proton gradient. The F_0_ subcomplex is composed of ATPF0A (K02108), ATPF0B (K02109), and ATPF0C (K02110), while the F_1_ subcomplex is composed of ATPF1A (K02111), ATPF1B (K02112), ATPF1D (K02113), ATPF1E (K02114), and ATPF1G (K02115). The genes encoding these subunits are usually arranged consecutively in a conserved genetic locus, which also includes another subunit, ATPI (K02116) and sometimes atpR. K02116 is interchangeably associated with two pfam domains, which makes orthologous gene assignments problematic: for consistency in the text below, ATPI sequences containing the pfam03899-ATP_synthI domain will be called “sI”, and ATPI sequences containing the pfam09527-ATPase_gene1 will be called “I”: atpR sequences containing the pfam12966-atpR domain will be called “R”.

**Figure 2:**
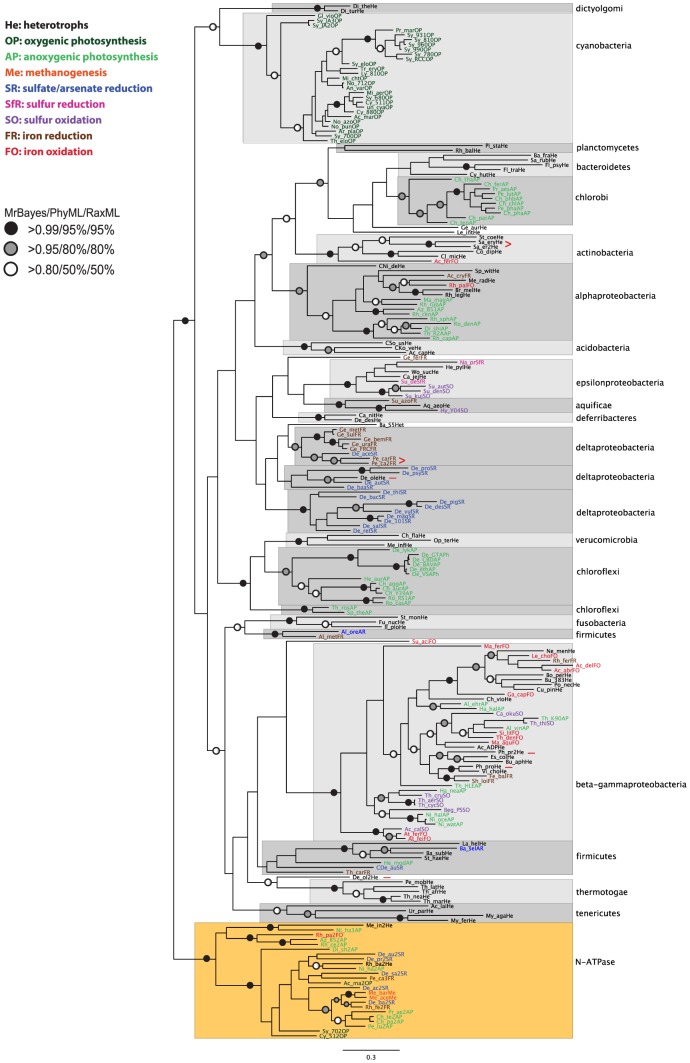
Phylogenetic reconstruction of ATPF0A. The tree shown is the best Bayesian topology, based on 215 sequences and 232 amino acid positions (length after trimming; median sequence length before trimming: 254). Numerical values at the nodes of the tree (x/y/z) indicate statistical support by MrBayes, PhyML and RAxML (posterior probability, bootstrap and bootstrap, respectively). Values for highly supported nodes have been replaced by symbols, as indicated. Species names are colour-coded based on their bioenergetic mode as in Figure 1. Full details and accession numbers for all protein sequences used are given in Table S1. The tree is rooted at the N-ATPase clade, previously reported to be the result of horizontal gene transfer in a variety of species, all of which also contain a canonical ATPF_0_F_1_ (apart from the two *Methanosarcina* species shown, which also have a canonical ATPV). The tree confidently separates the major bacterial taxonomic lineages, but with limited support for their branching order: strong support is provided for a subgroup containing the verrucomicrobia and chloroflexi, while another subgroup containing the alpha-proteobacteria, actinobacteria, chlorobi, bacteroidetes and planctomycetes also has reasonable support. This group also includes the spirochaete *Leptospira interrogans* and the gemmatimonadete *Gemmatimonas aurantiaca*, as well as *Candidatus Nitrospira defluvii* which groups with the alpha-proteobacteria. Reasonable support is also provided for the grouping of dictyoglomi and cyanobacteria, and for a subgroup containing the fusobacteria, firmicutes, tenericutes, thermotogae, and beta-gamma-proteobacteria. Two species-specific duplications (in *Saccharopolyspora erythraea* and *Pelobacter carbinolicus*) are highlighted with a red “>”. Two further duplications are highlighted with a red “-” after the species name; in *Photobacterium profundum* the duplication either occurred before the split from other closely-related species or represents HGT from other gamma-proteobacteria; the duplication in *Desulfococcus oleovorans* possibly represents HGT from thermotogae (also see Figures S1 and S2).

**Figure 3:**
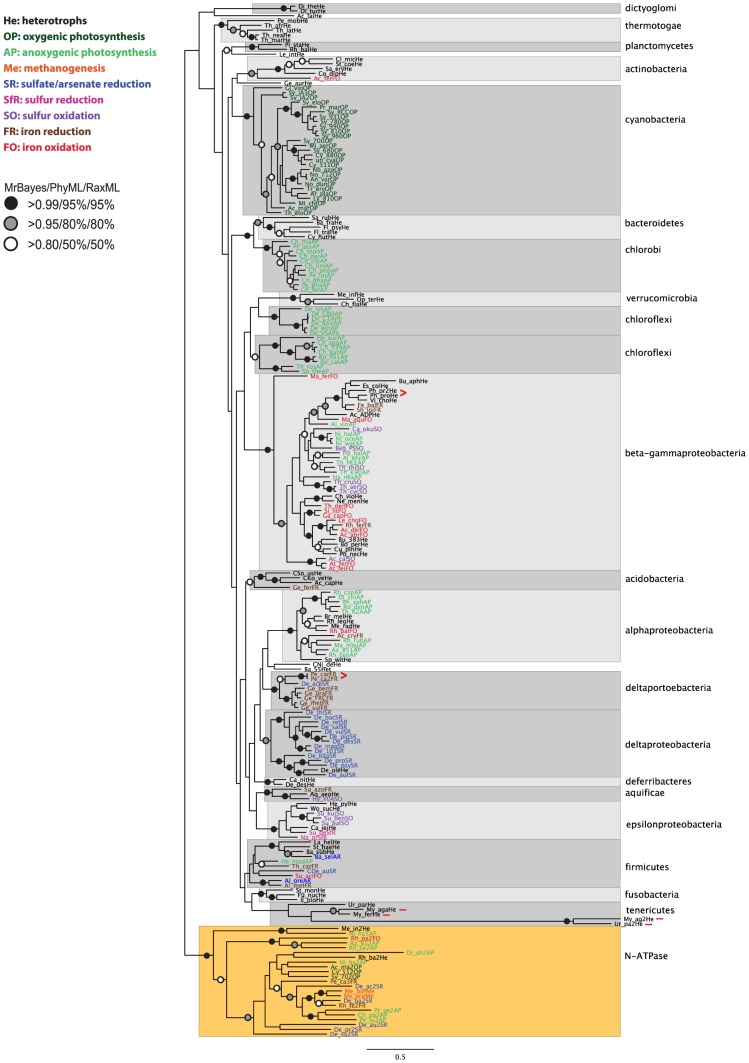
Phylogenetic reconstruction of ATPF1A. The tree shown is the best Bayesian topology, based on 215 sequences and 502 amino acid positions (length after trimming; median sequence length before trimming: 508). Numerical values at the nodes of the tree (x/y/z) indicate statistical support by MrBayes, PhyML and RAxML (posterior probability, bootstrap and bootstrap, respectively). Values for highly supported nodes have been replaced by symbols, as indicated. Species names are colour-coded based on their bioenergetic mode as in Figure 1. Full details and accession numbers for all protein sequences used are given in Table S1. The tree is rooted at the N-ATPase clade, previously reported to be the result of horizontal gene transfer in a variety of species, all of which also contain a canonical ATPF_0_F_1_ (apart from the two *Methanosarcina* species shown, which also have a canonical ATPV). The tree confidently separates the major bacterial taxonomic lineages, but with limited support for their branching order: reasonable support is only provided for one subgroup containing the chlorobi, and the bacteroidetes. The spirochaete *Leptospira interrogans* groups with the planctomycetes. Two species-specific duplications (in *Photobacterium profundum* and *Pelobacter carbinolicus*) are highlighted with a red “>”. Two further duplications within the tenericutes are highlighted with a red “-” after the species name; this duplication likely happened before the split between *Mycoplasma agalactiae* and *Ureaplasma parvum*.

For each subunit, the corresponding protein sequences were downloaded from KEGG for all species and, after multiple alignment, phylogenetic analysis was performed using Bayesian and maximum likelihood methods. The phylogenetic analysis for ATPF0A and ATPF1A are shown in Figures 2 and 3, respectively, while the rest of the trees are in Figures S1, S2, S3, S4, S5, S6, S7. Overall, for all subunits, species segregate based on taxonomic groups with good bootstrap support, as in the 16S tree, and not based on bioenergetic mode. If the current patchy distribution of bioenergetic modes (Figure 1) is due to HGT, we might expect the ATP synthase sequences from different organisms which utilize the same pathway to group together (as we used different colours for the different bioenergetic modes for species names on the tree, we would essentially expect to see organisms grouping based on colour). This is not what we observe, suggesting that there is no evidence of HGT of the ATP synthase despite the use of different bioenergetic modes between closely related species.

**Figure 4:**
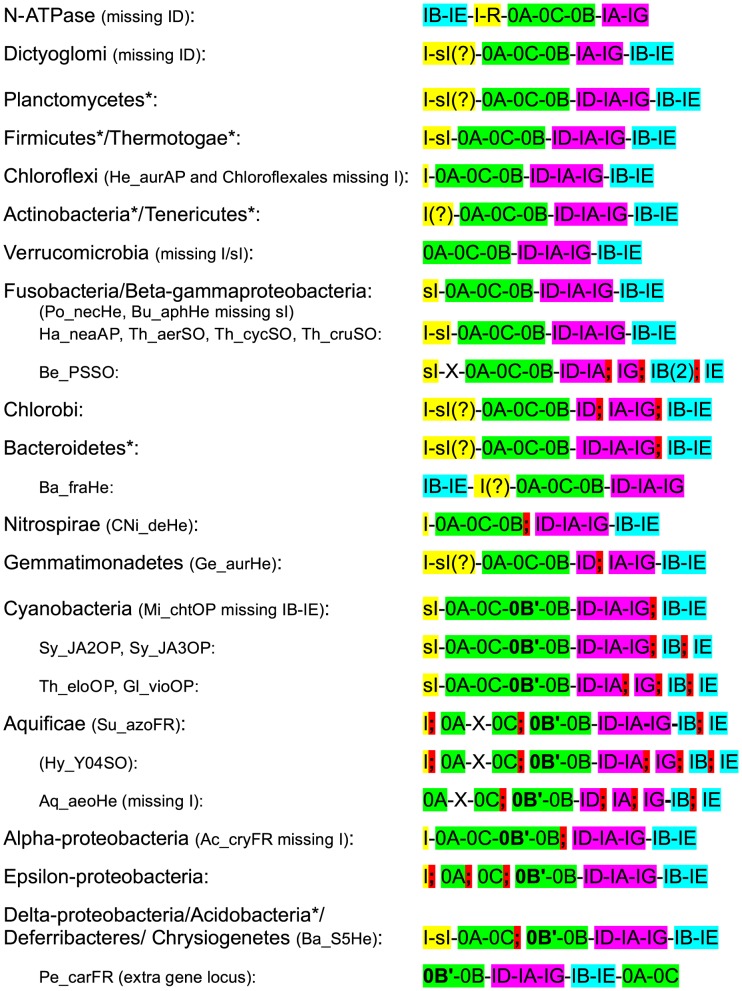
ATPF_0_F_1_ gene locus organization per lineage. The ATPF_0_F_1_ gene locus organization was checked for all species in the IMG database [47], and is summarized per lineage. The gene order shown follows the order in which the genes are transcribed in each genome (upstream to downstream). Semicolons indicate that the separated gene groups are on non-adjacent genetic locations (and can be very far upstream or downstream; e.g. separated by only 4 intervening ORFs in *Geobacter* sp. FRC-32, and by up to 5026 intervening ORFs, or 6 Mb, in *Nostoc* sp. PCC 7120; see Table S1). When the locus is split, the genes are shown in the order they are usually found in when the locus is intact. ATPF0B (K02109) is often duplicated, so one copy is called 0B, and the other 0B′, based on the gene order. ATPI (K02116) is also often duplicated, and is designated “I” “sI” and “R” based on the presence of distinct pfam domains, as discussed in the text. Question marks indicate that the ATPI subunit is sometimes not clearly assigned to the orthology group. “X” denotes hypothetical intervening ORFs. Notable variations within some lineages are shown. *Especially for lineages represented by relatively few species, please see TableS1 for variations between the species examined within each lineage.

Nevertheless, in certain species, a duplication of the whole ATPF0F1 locus is seen (Table 2), and the majority of those duplications correspond to the so-called N-ATPase, which appears to have been acquired via horizontal gene transfer, as has been reported previously [32]. The N-ATPase genetic locus is characterized by the absence of the ATPF1D gene and the presence of the *atpR* gene (Figure 4) as well as a long (>100aa) C-terminal extension in ATPF0B (Dataset S1). For the set of organisms studied here, the N-ATPase is found in certain species of planctomycetes (*Rhodopirellula baltica*), verrucomicrobia (*Methylacidiphilum infernorum*), chlorobi (*Chlorobaculum parvum*, *Chlorobaculum tepidum* (partial), *Pelodictyon luteolum*, *Prosthecochloris aestuarii*), cyanobacteria (*Acaryochloris marina*, *Cyanothece* sp. ATCC 51142, *Synechococcus* sp. PCC 7002), alpha-proteobacteria (*Azospirillum* sp. B510, *Dinoroseobacter shibae*, *Rhodopseudomonas palustris*, *Rhodospirillum centenum*/*Rhodocista centenaria*), beta-proteobacteria (*Rhodoferax ferrireducens*), gamma-proteobacteria (*Nitrosococcus halophilus* - double N-ATPase, one locus split, both missing atpR), delta-proteobacteria (*Desulfobacterium autotrophicum*, *Desulfobulbus propionicus*, *Desulfomicrobium baculatum*, *Desulfovibrio salexigens*, *Desulfuromonas acetoxidans*, *Pelobacter carbinolicus*) and methanomicrobia (*Methanosarcina acetivorans*, *Methanosarcina barkeri*).

**Table 2:** Gene duplications of ATP synthase subunits in the species analyzed.

Lineage	species	ATPF0A	ATPF0B	ATPF0C	ATPF1A	ATPF1B	ATPF1D	ATPF1E	ATPF1G	comments
γ-proteobacteria	Ph_proHe	LSD	LSD	LSD	SSD	SSD	LSD	SSD	LSD	double full locus
δ-proteobacteria	Pe_carFR	SSD & N-ATPase	SSD & N-ATPase	SSD & N-ATPase	SSD & N-ATPase	SSD & N-ATPase	SSD	SSD & N-ATPase	SSD & N-ATPase	double full locus & N-ATPase
δ-proteobacteria	De_oleHe	HGT? (Thermotogae)	LSD	HGT? (Thermotogae)	–	–	–	–	–	double OA-OC-OB′-OB
Actinobacteria	Sa_eryHe	SSD	–	–	–	–	–	–	–	double 0A (ectopic)
ζ-proteobacteria	Ma_ferFO	–	HGT? (Planctomycetes)	–	–	–	–	SSD	–	double IE; 0B (both ectopic)
Firmicutes	Al_metFR	–	–	SSD	–	–	–	–	–	double OC (in-locus)
Tenericutes	My_agaHe	–	–	–	LSD^a^	LSD^a^	–	–	–	double IA-IB (ectopic)
Tenericutes	Ur_parHe	–	–	–	LSD^a^	LSD^a^	SSD	–	–	double IA-IB (ectopic); double ID (in-locus)
β-proteobacteria	Th_denFO	–	–	–	–	–	–	LSD	–	double IE (ectopic)
γ-proteobacteria	Ac_calSO	–	–	–	–	–	–	LSD^b^	–	double IE (ectopic)
γ-proteobacteria	At_ferFO	–	–	–	–	–	–	LSD^b^ & SSD	N-ATPase	triple IE; double IG (all ectopic)
γ-proteobacteria	At_feiFO	–	–	–	–	–	–	LSD^b^	–	double IE (ectopic)
δ-proteobacteria	De_magSR	–	–	–	–	–	–	LSD^c^	–	double IE (in-locus)
δ-proteobacteria	De_101SR	–	–	–	–	–	–	LSD^c^	–	double IE (in-locus)
Aquificae	Aq_aeoHe	–	–	–	–	–	–	–	HGT? (Dictyoglomi)	double IG (ectopic)
α-proteobacteria	Az_B51AP	N-ATPase	x	N-ATPase	N-ATPase	N-ATPase	–	N-ATPase	N-ATPase	
α-proteobacteria	Di_shiAP	N-ATPase	N-ATPase	N-ATPase	N-ATPase	N-ATPase	–	N-ATPase	N-ATPase	
α-proteobacteria	Rh_cenAP	N-ATPase	N-ATPase	N-ATPase	N-ATPase	N-ATPase	–	N-ATPase	N-ATPase	
α-proteobacteria	Rh_palFO	N-ATPase	N-ATPase	N-ATPase	N-ATPase	N-ATPase	–	N-ATPase	N-ATPase	
β-proteobacteria	Rh_ferFR	N-ATPase	N-ATPase	N-ATPase	N-ATPase	N-ATPase	–	N-ATPase	N-ATPase	
γ-proteobacteria	Ni_halAP	N-ATPase(2)	N-ATPase(2)	N-ATPase(2)	N-ATPase(2)	N-ATPase(2)	–	N-ATPase(2)	N-ATPase(2)	2 N-ATPases, one is split in half
δ-proteobacteria	De_aceSR	N-ATPase	N-ATPase	N-ATPase	N-ATPase	N-ATPase	–	N-ATPase	N-ATPase	
δ-proteobacteria	De_autSR	N-ATPase	N-ATPase	N-ATPase	N-ATPase	N-ATPase	–	N-ATPase	N-ATPase	
δ-proteobacteria	De_bacSR	N-ATPase	N-ATPase	N-ATPase	N-ATPase	N-ATPase	–	N-ATPase	N-ATPase	
δ-proteobacteria	De_proSR	N-ATPase	N-ATPase	N-ATPase	N-ATPase	N-ATPase	–	N-ATPase	N-ATPase	
δ-proteobacteria	De_salSR	N-ATPase	N-ATPase	N-ATPase	N-ATPase	N-ATPase	–	N-ATPase	N-ATPase	
Chlorobi	Ch_tepAP	N-ATPase	–	–	–	N-ATPase	–	N-ATPase	–	partial N-ATPase locus
Chlorobi	Ch_parAP	N-ATPase	N-ATPase	N-ATPase	N-ATPase	N-ATPase	–	N-ATPase	N-ATPase	
Chlorobi	Pe_lutAP	N-ATPase	N-ATPase	N-ATPase	N-ATPase	N-ATPase	–	N-ATPase	N-ATPase	
Chlorobi	Pr_aesAP	N-ATPase	N-ATPase	N-ATPase	N-ATPase	N-ATPase	–	N-ATPase	N-ATPase	
Cyanobacteria	Ac_marOP	N-ATPase	N-ATPase	N-ATPase	N-ATPase	N-ATPase	–	N-ATPase	N-ATPase	
Cyanobacteria	Cy_511OP	N-ATPase	N-ATPase	N-ATPase	N-ATPase	N-ATPase	–	N-ATPase	N-ATPase	
Cyanobacteria	Sy_700OP	N-ATPase	N-ATPase	N-ATPase	N-ATPase	N-ATPase	–	N-ATPase	N-ATPase	
Planctomycetes	Rh_balHe	N-ATPase	N-ATPase	N-ATPase	N-ATPase	N-ATPase	–	N-ATPase	N-ATPase	
Verrucomicrobia	Me_infHe	N-ATPase	N-ATPase?	N-ATPase	N-ATPase	N-ATPase	–	N-ATPase	N-ATPase	
	duplicates	24	90*	23	23	24	3	29	23	
	triplicates	2	14*	2	2	2	–	3	2	

LSD = lineage-specific duplication (before split from closely related species).

SSD = species-specific duplication.

HGT = horizontal gene transfer/extremely divergent.

– = not duplicated.

a = duplication before split between My_agaHe & Ur_parHe (see text, Figure 4 and Figure S3).

b = duplication before split between Ac_calSO, At_ferFO & At_feiFO (see text and Figure S5).

c = duplication before split between De_magSR & De_101SR (see text and Figure S5).

x = missing (probably misannotated as part of ATPF1A).

*  = most correspond to ancient 0B′ duplication (see text and Figure S1).

The sequences corresponding to the N-ATPase form a highly supported monophyletic group; the trees (apart from ATPF1D) were therefore rooted at this N-ATPase clade. Phylogenetic reconstruction of all subunits confidently separates the major bacterial taxonomic lineages, but the trees only give limited support for the branching order (Figures 2–3, S1, S2, S3, S4, S5, S6). The differences between trees, with respect to the resolution of the branching order of different lineages, are probably due to the sequence length of the proteins analyzed; longer subunits retain more information and tend to give better-resolved phylogenetic trees, than shorter sequences [33]. The most clear-cut grouping is that of the beta- and gamma-proteobacteria, which is seen in all trees, and has significant bootstrap support in all but the ATPF1D and ATPF1E trees. Significant bootstrap support for the beta- and gamma-proteobacteria grouping is also seen in the 16S phylogenetic analysis (Figure 1), which also suggests groupings of the chlorobi and the bacteroidetes, and of the fusobacteria and tenericutes. The phylogenetic link between the chlorobi and the bacteroidetes is also seen in the trees for ATPF0A (Figure 2), ATPF0C (Figure S2), ATPF1A (Figure 3) and ATFP1B (Figure S3). In the ATPF0C analysis this group also includes the planctomycetes as well as the spirochaete *Leptospira interrogans* and the gemmatimonadete *Gemmatimonas aurantiaca* (*Leptospira interrogans* also groups with the planctomycetes in the ATPF1A phylogeny). The ATPF0A phylogeny also has reasonable support for grouping the chlorobi, bacteroidetes and planctomycetes, together with the actinobacteria and the alpha-proteobacteria (this group also includes the spirochaete *Leptospira interrogans* and the gemmatimonadete *Gemmatimonas aurantiaca*, as well as *Candidatus Nitrospira defluvii* which groups with the alpha-proteobacteria; *Candidatus Nitrospira defluvii* also groups with the alpha-proteobacteria in the ATPF0C analysis). A group containing the actinobacteria and the planctomycetes (as well as the spirochaete *Leptospira interrogans* and the gemmatimonadete *Gemmatimonas aurantiaca*) is supported by the ATPF1G tree. Strong support is provided by the ATPF0A phylogeny for a group containing the verrucomicrobia and chloroflexi; the phylogenetic reconstruction of ATPF1G (Figure S6) also has reasonable support for a group containing the verrucomicrobia, chloroflexi, and the beta-gamma-proteobacteria. Finally, reasonable support is provided in the ATPF0A tree for the grouping of dictyoglomi and cyanobacteria, and for a group containing the fusobacteria, firmicutes, tenericutes, thermotogae, and beta-gamma-proteobacteria. In the ATPF0C analysis, the dictyglomi cluster with the N-ATPase with good statistical support (Figure S2).

Although the phylogenetic analysis is based on trimmed sequences, i.e. only the unambiguous homologous regions were retained for phylogenetic analysis by manually inspecting and masking/trimming the sequences, some notable insertions/deletions were noted in the multiple alignments. For example, the chlorobi and the bacteroidetes are both missing the C-terminal half of ATPF1E, and share an internal 10–15aa insertion in ATPF1A. A different internal 10–15aa insertion in ATPF1A is shared between the beta- and gamma-proteobacteria. Actinobacteria have a ∼75aa insertion near the N-terminus of ATPF1D, and cyanobacteria have an internal 20aa insertion in ATPF1G. The N-ATPase ATPF1A in *Azospirillum* sp. B510 has a long (∼100aa) N-terminal extension plus a ∼150aa insertion near the N-terminus, while the N-ATPase ATPF1G in *Cyanothece* sp. ATCC 51142 has a 50aa N-terminal extension (Dataset S1). The elucidation of the role of these signature sequences would require further study based on experimental or structural analysis.

### Genetic locus organization of the ATP synthase genes

Given the ancient origin of the ATP synthase complex, the syntenic genetic location of the genes was checked in all lineages, to identify common gene order transversions, gene duplications, and possible horizontal gene transfer events (Figure 4). The N-ATPase, which has been suggested to be an early-diverging branch of membrane ATPases [32] has the following gene order: IB-IE-I-R-0A-0C-0B-IA-IG. *Bacteroides fragilis* also has a similar gene locus organization, except that it lacks *atpR*. The subunits are arranged in consecutive order (i.e. the locus is not split) in the dictyoglomi, planctomycetes, firmicutes, thermotogae, chloroflexi, actinobacteria, tenericutes, verrucomicrobia, fusobacteria and the beta- and gamma-proteobacteria. Except for the proteobacteria and the verrucomicrobia, these lineages have been suggested to be near the base of the bacterial clade, either based on phylogenetic analysis [25], [31] or based on the analysis of signature sequences [26], [30]. By inference, the most likely ancient gene order for the ATPF_0_F_1_ locus is: I-sI-0A-0C-0B-ID-IA-IG-IB-IE, although some lineages lack I or sI or both (e.g. fusobacteria, chloroflexi, verrucomicrobia).

The locus has been split (indicated by semi-colons in Figure 4) at the junction between IG and IB in the chlorobi, bacteroidetes, cyanobacteria, aquificae and *Beggiatoa*, with further splits between IB and IE in aquificae and *Beggiatoa*. A further split is seen between ID and IA in the chlorobi and between IA and IG in aquificae and *Beggiatoa*. A split between 0B and ID is seen in nitrospirae and the alpha-proteobacteria, while a split between 0C and 0B is seen in aquificae, acidobacteria, deferribacteres, and delta- and epsilon-proteobacteria. A split between 0A and 0C has occurred in the epsilon-proteobacteria. Finally a split between I and OA is seen in aquificae. Therefore, although there are three “blocks” of genes which are usually conserved, in terms of gene order (one containing I(-sI)-0A-0C(-0B′)-0B, another containing ID-IA-IG, and another with IB-IE), in principle, gene-order transversion can and has happened all along the genetic locus.

### Most commonly duplicated/lost genes

The phylogenetic analysis and the gene locus information were used to examine the most likely origin of duplicated genes, i.e. whether they arose as gene duplications within a particular species, or via horizontal gene transfer (Table 2). In the delta-proteobacterium *Pelobacter carbinolicus*, there are two duplications of the whole ATPF_0_F_1_ locus, one corresponds to the N-ATPase, and the other is a species-specific duplication (Figures 2–3, S1, S2, S3, S4, S5, S6). A duplicated ATPF_0_F_1_ full locus, which does not correspond to the N-ATPase was also found in the gamma-proteobacterium *Photobacterium profundum*; this appears as a species-specific duplication in the ATPF1A, ATPF1B and ATPF1E trees (Figures 3, S3, S5), while in the rest of the trees, one copy groups with *Vibrio cholerae* and the other elsewhere within the gamma clade (Figure 2, S1, S2, S4, S6). This possibly hints at HGT from another closely related species, but the placement within the gamma clade is not consistent and could thus simply be due to high sequence divergence of the second copy in *P. profundum* for some of the subunits.

There are also certain *in-locus* gene duplications, where the duplicated genes are still found adjacent to each other on the genetic locus, as well as ectopic duplications (outside the main ATPF_0_F_1_ locus) probably resulting from recombinations/transversions (summarized in Table 2). The most commonly *in-locus* duplicated genes are ATPF0B and ATPI, discussed in more detail in the next section. The delta-proteobacterium *Desulfococcus oleovorans* has a fully duplicated ectopic ATPF0 complement; in the ATPF0B phylogeny (Figure S1) both copies group within the delta-proteobacteria suggesting that this could be a species-specific duplication where one copy has diversified. However, the duplicated ATPF0A (Figure 2) and ATPF0C (Figure S2) subunits group with the thermotogae with good bootstrap support, hinting at a possible HGT event; assuming a common origin for all three subunits in the duplicated locus, this suggestion of HGT from *Thermotogae* requires further study (phylogenetic analysis of only the deltaproteobacteria and thermotogae sequences did not resolve this issue as it gives the same results as above for the duplicated subunits, data not shown). The actinobacterium *Saccharopolyspora erythraea* has a duplicated ectopic ATPF0A, which looks like a species-specific duplication (Figure 2). The zeta-proteobacterium *Mariprofundus ferrooxydans* has a duplicated ectopic ATPF0B; it is unclear if this is the result of HGT, as the sequence groups with planctomycetes, but not with high bootstrap support (Figure S1). The firmicute *Alkaliphilus metalliredigens* has a species-specific *in-locus* duplication of ATPF0C (Figure S2) which is characterized by a long (∼100aa) N-terminal extension (Dataset S1). Ectopic duplications of ATPF1A and ATPF1B are seen in *Mycoplasma agalactiae* and *Ureaplasma parvum* (tenericutes) as has been reported recently [34]; this duplication likely happened before the split between the two species (Figure 3, S3); one of the ATPF1A copies in *U. parvum* has a long (∼250aa) C-terminal extension (Dataset S1). *Ureaplasma parvum* also has duplicated ATPF1D *in-locus*; the evolutionary history of this duplication cannot be clearly inferred from the phylogenetic analysis, although it appears to be species-specific in the PhyML and RaxML trees, but is not statistically supported by high bootstrap values. ATPF1E is duplicated ectopically in *Mariprofundus ferrooxydans* (zeta-proteobacteria), *Thiobacillus denitrificans* (beta-proteobacteria), and the gamma-proteobacteria *Acidithiobacillus caldus*, *Acidithiobacillus ferrivorans*, and *Acidithiobacillus ferrooxidans* (two extra copies), as well as *in-locus* in the delta-proteobacteria *Desulfovibrio magneticus* and *Desulfovibrio* sp. FW1012B. The duplication in *M. ferrooxydans* is species-specific, while the other duplications are lineage-specific, i.e. the duplication either occurred before the split from other closely-related species or represents HGT from other closely-related species (Figure S5): the duplication in *T. denitrificans* may represent HGT from other gamma-proteobacteria; a duplication occurred before the split between the gamma-proteobacteria *Acidithiobacillus ferrooxidans*, *Acidithiobacillus ferrivorans* and *Acidithiobacillus caldus*, with a further species-specific duplication in *Acidithiobacillus ferrooxidans*; another duplication occurred before the split between *Desulfovibrio magneticus* and *Desulfovibrio* sp. FW1012B in the delta-proteobacteria. ATPF1G is ectopically duplicated in *Aquifex aeolicus* (aquificae; one copy has a 80aa C-terminal extension) and *Acidithiobacillus ferrooxidans* (gamma-proteobacteria; one copy is missing the N-terminal half); the duplication in *Aquifex aeolicus* represents a very divergent sequence which groups with the dictyoglomi in the MrBayes and PhyML trees (Figure S6); the duplication in *A. ferrooxidans* might be a pseudogene as it is much smaller in size - in the tree it clusters with the N-ATPase genes.

ATPF0B is duplicated *in-locus* in acidobacteria, aquificae, cyanobacteria, deferribacteres, and alpha- delta- and epsilon-proteobacteria. This raises the question of whether ATPF0B has been duplicated independently in separate lineages, or whether the duplication has been passed on, either by direct descent, or by horizontal gene transfer. In the phylogenetic analysis (Figure S1) the ATPF0B′ group in the alpha-proteobacteria appears as a sister group to the alpha-proteobacterial ATPF0B, but with only moderate statistical support (red asterisk: 0.7 posterior probability in MrBayes, 50%, and 46% bootstrap support in PhyML and RaxML, respectively). The other ATPF0B's group together (blue asterisk), with good statistical support by MrBayes (posterior probability: 1) but with low support in PhyML and RaxML (24% and 26% bootstrap support, respectively). The grouping of the alpha-proteobacterial ATPF0B and ATPF0B′ may indicate that this duplication happened more recently than the ATPF0B duplications in the other lineages. However, given the low bootstrap support it remains unclear from the tree whether the ATPF0B/0B′ duplication happened independently in the different lineages where it is observed, or whether it happened only once in the common ancestor of all the lineages where it is observed (and presumably lost in other lineages, e.g. the beta-gamma-proteobacteria); however, the latter scenario is more plausible based on parsimony considerations.

Notable absences are the ATPF1D in N-ATPase, as well as in dictyoglomi (*Dictyoglomus thermophilum* and *Dictyoglomus turgidum*), ATPF0C in *Wolinella succinogenes* (epsilon-proteobacteria), ATPF1B and ATPF1E in the cyanobacterium *Microcoleus chthonoplastes*, and ATPI missing from many species (e.g. chloroflexales, verrucomicrobia). At least some of these absences may of course be due to incomplete annotation or extreme sequence divergence.

### Evolution of atpI, sI, and R

ATPI has been the least studied subunit of the F_0_F_1_ ATP synthase complex. As mentioned above, ATPI (K02116) is interchangeably associated with two pfam domains (pfam03899-ATP_synthI and pfam09527-ATPase_gene1), which makes orthologous gene assignments problematic. The bacterial *uncI* gene encoding a small transmembrane protein which includes the pfam03899 domain, has been demonstrated to have a chaperone role in assisting the assembly of the c-ring of the F_0_ subcomplex [35], [36]. By analogy, it has been suggested that the *atpR* gene of the N-ATPase (characterized by the presence of the pfam12966 domain) plays a similar role, in the absence of *uncI* [32]. Given this suggestion, and the grouping of the *atpQ* genes (which include the pfam09527 domain) into the same KEGG cluster as *uncI*, along with the fact that all three encode proteins of similar size and, based on their position in the genetic cluster, could be the result of gene duplications, we decided to analyze their evolutionary relationship in more detail.

The phylogenetic reconstruction of ATPI (K02116) protein sequences, including “sI” proteins containing the pfam03899-ATP_synthI domain, “I” proteins containing the pfam09527-ATPase_gene1, and “R” proteins containing the pfam12966-atpR domain (found in the N-ATPase locus) is shown in Figure S7. Overall the three types of proteins look similar in the alignment, although atpR stands out, as do the cyanobacterial sI sequences; the delta-proteobacterium *Desulfovibrio piger* has a prominent 50aa C-terminal extension (Dataset S1). Only the PhyML tree is shown, even though the bootstrap support for most branches is not significant. Phylogenetic analysis with the same set of sequences using MrBayes failed to converge on a tree, and the RaxML tree had very bad resolution. The low resolution and low bootstrap support are probably due to the short sequence length and high divergence of these sequences. Nevertheless, the tree does separate a cluster of the “I” proteins (which contain the pfam09527-ATPase_gene1 domain) to the left of the dotted grey line, and another cluster containing the “sI” proteins (pfam03899-ATP_synthI domain) and “R” proteins (pfam12966-atpR domain) to the right of the grey dotted line. Based on the gene locus organization and the protein sizes, the genes encoding the “sI” and “R” proteins look like duplications of the “I” gene, and the tree indeed supports this hypothesis. However, due to the low resolution of the phylogenetic analysis, the issue of the origin and functional homology of atpI, sI, and R would ultimately need to be resolved with structural and functional analysis.

## Discussion

Phylogenetic analysis of 16S rRNA for 272 species chosen to represent all the major prokaryotic lineages and bioenergetic modes indicated that, overall, there is no monophyly of bioenergetic modes (one notable exception is oxygenic photosynthesis which is confined to the cyanobacteria). This analysis also highlighted lineages which include species with vastly different modes of generating energy (e.g. proteobacteria, firmicutes). The scattered distribution of certain bioenergetic modes, such as anoxygenic photosynthesis or iron oxidation, indicates rampant HGT of at least some bioenergetic modes, in agreement with previous analyses [16], [17], [18]. All these bioenergetic pathways also include the ATP synthase complex, but phylogenetic analysis of all the ATPF_0_F_1_ synthase subunits, common to almost all bacterial lineages, largely agree with the 16s rRNA tree. This indicates that, if different bioenergetic pathways dispersed into different lineages by horizontal gene transfer, this did not involve the ATP synthase complex. Presumably, each species used its pre-existing ATP synthase complex and adapted it to utilize the proton gradient generated by vastly different ETCs. Recent data has shown that large-scale HGT from bacteria transformed the bioenergetic capabilities of the Haloarchaea [37] and yet Haloarchaea retain ATPV, whereas their laterally acquired bioenergetics modes utilize ATPF in the bacteria. This is in agreement with our results, and again indicates flexibility in combining a species' pre-existing ATP synthase with a newly acquired electron transport chain. Given the widespread effect of HGT on prokaryotic evolution [38], [39], [40], it may be that the cost of incorporating a laterally transferred ATP synthase to replace a pre-existing enzyme is too high to overcome [41]. To our knowledge the question of whether specific modifications are needed for the ATP synthase to function with different bioenergetic modes has not been addressed previously, so this current, updated large-scope study allows us to resolve this issue, and suggests that no apparent such modifications exist, at least at the sequence level. A more thorough structural analysis would be needed to examine if certain structural modifications unite the ATP synthases of organisms using each bioenergetic pathway.

HGT has happened however, for a variant form of the ATP synthase, previously named N-ATPase, as it includes residues in the c subunit for translocating Na^+^ [32]. This is found always in addition to the F_0_F_1_ ATP synthase, in certain species from different bacterial lineages, as well as in two *Methanosarcina* species of the archaea. The N-ATPase subunits always cluster independently of their F_0_F_1_ counterparts, and although they often group closest to the dictyoglomi, only the ATPF0C phylogeny has significant bootstrap support for a grouping of the dictyoglomi and N-ATPase; therefore, their exact origin cannot be inferred from the tree, and possibly predates the separation between ATPV and ATPF [32]. The N-ATPase locus is characterized by the absence of the ATPF1D subunit, and the presence of the atpR gene (also see below). Interestingly, the two dictyoglomi species studied here (the only two for which complete genome information is available) also lack the ATPF1D subunit, which in combination with the close affinity of the dictyoglomi and the N-ATPase in most of the trees, might suggest that the dictyoglomi are the closest relative to the common ancestor of the N-ATPase. In the gamma-proteobacterium *Nitrosococcus halophilus*, two copies of the N-ATPase are found (one locus is split in half, both are missing *atpR*), whereas *Chlorobaculum tepidum* of the chlorobi only has half the locus; the lack of certain subunits may indicate a non-functional degenerate N-ATPase. It is assumed that the N-ATPase confers a selective advantage in high-salt environments [32].

Given the ancient origin of the F_0_F_1_ ATPase, the phylogenetic trees can perhaps give clues as to the evolutionary relationships between different bacterial lineages. The branching order of bacterial lineages remains an issue unresolved through phylogenetic analysis [25], [27], [28], [29], although other methods have also been proposed based on signature sequences of certain crucial proteins [26], and a more recent analysis based on feature frequency profiles in whole proteome data has produced a well-resolved tree [30]. Some of the F_0_F_1_ ATP synthase subunits are relatively long proteins, and relatively slow evolving due to their interactions with the other subunits, so they may retain some of the evolutionary signal that cannot be retrieved from 16S rRNA sequences. There is consistent support for a grouping of the beta- and gamma-proteobacteria, another of the chlorobi and the bacteroidetes, and some support for this group also including the planctomycetes, the actinobacteria, the alpha-proteobacteria and the spirochaete *Leptospira interrogans* and the gemmatimonadete *Gemmatimonas aurantiaca*; *Candidatus Nitrospira defluvii* groups with the alpha-proteobacteria. Some trees also indicate a subgroup containing the verrucomicrobia and the chloroflexi, and possibly also the beta-gamma-proteobacteria. Finally, reasonable support is provided in the ATPF0A tree for the grouping of dictyoglomi and cyanobacteria, and for a subgroup containing the fusobacteria, tenericutes, firmicutes, thermotogae, and beta-gamma-proteobacteria. The groupings of (i) the beta-gamma proteobacteria, (ii) the chlorobi and bacteroidetes, and (iii) the fusobacteria, tenericutes, firmicutes, and thermotogae, are in agreement with the more recent phylogeny [30].

The order of the genes encoding the F_0_F_1_ ATP synthase subunits is relatively well conserved overall in most of the species analyzed, although the locus has been split on multiple occasions, and the genes for ATPF1B and ATPF1E are found either upstream (in the N-ATPase and in *Bacteroides fragilis*) or, most commonly, downstream of all the others. Duplications of each of the F_0_F_1_ ATP synthase subunits are observed in several species, either within the genetic locus or in distant parts of the genome. The history of these duplications can be traced by looking at the phylogenetic analysis. The most ancient *in-locus* duplication is likely that of atpI, with the diversification of the downstream copy into “sI” and “R”, but with multiple losses in various lineages of either one or both copies. Another ancient *in-locus* duplication is that of ATPF0B, which probably occurred in the common ancestor of the acidobacteria, aquificae, cyanobacteria, deferribacteres, delta- epsilon- and alpha-proteobacteria, (and presumably lost in other lineages, e.g. the beta-gamma-proteobacteria). Most of the other duplications have occurred in isolated species, and appear to be species-specific, with no unassailable evidence of HGT.

These duplications raise the question of how certain species deal with gene dosage effects, e.g. to co-ordinate the ATP synthase complex structure. As there is no clear evidence of HGT, apart from the N-ATPase clade, most duplications seem to be the result of stochastic events that have not been bred out; presumably this means that at least some of these duplications, e.g. the ATPF0B/0B′ duplication may confer a selective advantage, although this would need to be confirmed experimentally. A recent study of the ATPV complex showed that such paralogous expansions can lead to increased complexity (and possibly also specificity) of a multi-subunit molecular machine [42]. Moreover, ATPF0B functions as a dimer, even in species where only one copy exists in the genome, and the two parts of the dimer interact with different parts of the F_1_ and the F_0_ subcomplex [43], [44]. Thus a gene duplication which allows each gene copy to fine-tune specific interactions may indeed be advantageous. Notably, cyanobacterial ATPF0B/0B′ have been successfully inserted into a null *E. coli* strain (which lacks its native single ATPF0B) and form heterodimers which assemble with the rest of the native ATP synthase *E. coli* subunits to form a functional enzyme [45]. This again points to a flexibility of the ATP synthase in different species, to accommodate changes and duplications.

The loss of ATPF1D from N-ATPase and dictyoglomi, as well as ATPI from many species also raises the question of the essentiality of these subunits for the function of the F_0_F_1_ ATP synthase. The absence of certain subunits in isolated species (ATPF0C from *Wolinella succinogenes* (epsilon-proteobacteria), ATPF1B and ATPF1E from the cyanobacterium *Microcoleus chthonoplastes*) may be due to incomplete annotation or extreme sequence divergence, although if they represent true losses, again this raises questions as to the functionality of the ATP synthase in these species.

Overall, this analysis highlights the patchy distribution of bioenergetic modes across prokaryotic lineages, which is most likely the result of HGT. However, there is no evidence of HGT for the ATP synthase to accompany the spread of bioenergetic pathways in different lineages. This means that the ATP synthase cannot be used to reconstruct the origin of the diversity of bioenergetic modes in prokaryotes. It also indicates that there are no apparent specific modifications of the F_0_F_1_ ATP synthase in order for it to work with different bioenergetic ETCs. The F_0_F_1_ ATP synthase genetic locus is overall well conserved, although as demonstrated by multiple splits and duplications, in principle, the system is robust and flexible, as it can deal with a split between any subunits and/or a duplication of any subunit. The elucidation of the way in which certain species deal with these duplications, splits and losses, and the advantage any of these may confer, now requires further study.

## Materials and Methods

### Organism selection

Bacteria and archaea species, whose genomes have been completely sequenced and are available at NCBI, were chosen by parsing the NCBI Genome Project database (http://www.ncbi.nlm.nih.gov/bioproject) with keywords relating to the relevant metabolisms (e.g. “anoxygenic phototroph”), and the relevant phyla (e.g. “chlorobi”). For autotrophs and chemolithotrophs, all relevant species were examined, but for heterotrophs, only a sample of species was examined so as to cover the full diversity of bacteria and archaea [31] (http://tolweb.org/tree/) and the full bioenergetic diversity per lineage. For lineages with many sequenced genomes, the tree of [31] was used to pick species so as to cover as much phylogenetic diversity as possible with a limited number of species. The set of species selected, represent 131 clusters, with a genome similarity score (GSS) threshold of 0.5; of those, 24 are in “clusters” which only have one member, and 63 are the sole representatives from their cluster [46]. Information on the metabolic mode of all species was also cross-checked in the IMG database [47]. Each species name was assigned an 8-character abbreviation for better data handling during the phylogenetic analysis, by keeping the first two letters of the first name and the first three letters of the second name, as well as a 2–3 letter ending, denoting the bioenergetic mode. Details of all the 272 organisms analyzed, and of the species names abbreviations are given in Table S1.

### Sequence retrieval and phylogenetic analysis

16S rRNA sequences were downloaded pre-aligned from the RDP database [48]. When more than one sequence was available for each species/strain examined, one of the good-quality >1200 bp sequences was selected at random, unless the type sequence was available, in which case that was selected. Importantly, we used data from the same strain for the 16S analysis and the ATP analysis (see below). As bacterial and archaeal sequences are provided as separate pre-aligned files, the program opal was used to align the two sets [49]. Common gaps were removed after manual examination of the whole set of sequences in McClade. The nucleotide substitution model that best fits the data (GTR+I+G) was selected using the program ModelGenerator [50] (http://bioinf.nuim.ie/modelgenerator/).

All other analyses were done at the amino acid level. For the ATP synthase subunits, sequence accession numbers were retrieved using the ortholog tables from the KEGG database: KEGG ortholog tables are based on RefSeq annotations, sequence similarity and best-hit searches, as well as tools for operon-like consistency and completeness of pathway modules and complexes; furthermore they are regularly updated (http://www.kegg.jp/kegg/ko.html). In cases where data was missing from the KEGG database, this was supplemented by data from IMG [47], manual analysis to find the best reciprocal BLAST hits, as well as synteny considerations, since the gene order of the ATP synthase locus is well-conserved overall. The accession numbers of all sequences analyzed, and the corresponding species names abbreviations, are given in Table S1. Sequences were downloaded from KEGG in fasta format using a custom perl script. Alignments were created using MUSCLE [51]. Only unambiguous homologous regions were retained for phylogenetic analysis by manually inspecting and masking/trimming the sequences in McClade (the masked alignment are given in Dataset S1). ProtTest [52] was used to estimate the appropriate model of sequence evolution.

Phylogenetic analysis was performed by three separate methods. To obtain the Bayesian tree topology and posterior probability values, the program MrBayes version 3.1.2 was used [53]. Analyses were run for 1–5 million generations, removing all trees before a plateau established by graphical estimation. All calculations were checked for convergence and had a splits frequency of <0.1. Maximum-likelihood (ML) analysis was performed using PhyML [54] and RAxML [55] with 100 bootstrap replicates. Nodes with better than 0.95 posterior probability and 80% bootstrap support were considered robust, and nodes with better than 0.80 posterior probability and 50% bootstrap support are shown. Tree files were processed in Figtree v1.4 and Adobe Illustrator to highlight homologous groups, and colour-code species names based on bioenergetic mode.

### Genetic locus analysis

As the genes encoding the different subunits of the ATP synthase are normally clustered in an operon, the genetic locus of the sequences analyzed was examined in the IMG database [47]. Details of the locus organization in each species are given in Table S1 and the data is summarized per lineage in Figure 4.

## Supporting Information

Figure S1 **Phylogenetic reconstruction of ATPF0B.** The tree shown is the best Bayesian topology, based on 298 sequences and 161 amino acid positions (length after trimming; median sequence length before trimming: 170). Numerical values at the nodes of the tree (x/y/z) indicate statistical support by MrBayes, PhyML and RAxML (posterior probability, bootstrap and bootstrap, respectively). Values for highly supported nodes have been replaced by symbols, as indicated. Species names are colour-coded based on their bioenergetic mode. Full details and accession numbers for all protein sequences used are given in Table S1. The tree is rooted at the N-ATPase clade, previously reported to be the result of horizontal gene transfer in a variety of species, all of which also contain a canonical ATPF_0_F_1_ (apart from the two *Methanosarcina* species shown which also have a canonical ATPV). The tree confidently separates the major bacterial taxonomic lineages, but with no clear support for their branching order; notably however, the dictyglomi cluster with the N-ATPase with good statistical support. There are multiple duplications, most of which represent the *in-locus* duplication of ATPF0B/0B′ seen in acidobacteria, aquificae, cyanobacteria, deferribacteres, delta- epsilon- and alpha-proteobacteria. The ATPF0B′ group in the alpha-proteobacteria appears as a sister group to the alpha-proteobacterial ATPF0B, although with only moderate statistical support (red asterisk: 0.7 posterior probability in MrBayes, 50%, and 46% bootstrap support in PhyML and RaxML, respectively). The other ATPF0B's group together (blue asterisk), with good statistical support by MrBayes (posterior probability: 1) but with low support by PhyML and RaxML (24% and 26% bootstrap support, respectively). It is thus unclear from the tree whether the ATPF0B/0B′ duplication happened independently in the different lineages where it is observed, or whether it happened only once in the common ancestor of all the lineages where it is observed (and presumably lost in other lineages, e.g. the beta-gamma-proteobacteria); however, the latter scenario is more plausible based on parsimony considerations. Two species-specific duplications in *Pelobacter carbinolicus* are highlighted with a red “>”. Four more duplications are highlighted with a red “-” after the species name: in *Photobacterium profundum* the duplication either occurred before the split from other closely-related species or represents HGT from other gamma-proteobacteria; in *Desulfococcus oleovorans* the duplication (which is a duplication of the full ATPF0 locus) seems to be species-specific, contrary to what is seen for ATPF0A in Figure 2 and ATPF0C in Figure S2; it is unclear if the duplication in the zeta-proteobacterium *Mariprofundus ferrooxydans* is the result of HGT, as the sequence groups with planctomycetes, but not with high bootstrap support. The duplication in *Methylacidiphilum infernorum* (highlighted with a yellow “-” after the species name) represents the ATPF0B within the N-ATPase locus, but it did not group with the other N-ATPase ATPF0Bs, probably due to its long branch length.
(EPS)

Figure S2 **Phylogenetic reconstruction of ATPF0C.** The tree shown is the best Bayesian topology, based on 214 sequences and 77 amino acid positions (length after trimming; median sequence length before trimming: 81). Numerical values at the nodes of the tree (x/y/z) indicate statistical support by MrBayes, PhyML and RAxML (posterior probability, bootstrap and bootstrap, respectively). Values for highly supported nodes have been replaced by symbols, as indicated. Species names are colour-coded based on their bioenergetic mode. Full details and accession numbers for all protein sequences used are given in Table S1. The tree is rooted at the N-ATPase clade, previously reported to be the result of horizontal gene transfer in a variety of species, all of which also contain a canonical ATPF_0_F_1_ (apart from the two *Methanosarcina* species shown which also have a canonical ATPV). The tree confidently separates the major bacterial taxonomic lineages, but with limited support for their branching order: reasonable support is only provided for one subgroup containing the chlorobi, bacteroidetes and planctomycetes (as well as the spirochaete *Leptospira interrogans* and the gemmatimonadete *Gemmatimonas aurantiaca*). *Candidatus Nitrospira defluvii* groups with the alpha-proteobacteria. Two species-specific duplications (in *Pelobacter carbinolicus* and *Alkaliphilus metalliredigens*) are highlighted with a red “>”. Two further duplications are highlighted with a red “-”after the species name; in *Photobacterium profundum* the duplication either occurred before the split from other closely-related species or represents HGT from other gamma-proteobacteria; the duplication in *Desulfococcus oleovorans* possibly represents HGT from thermotogae (also see Figure 2).
(EPS)

Figure S3 **Phylogenetic reconstruction of ATPF1B.** The tree shown is the best Bayesian topology, based on 215 sequences and 458 amino acid positions (length after trimming; median sequence length before trimming: 470). Numerical values at the nodes of the tree (x/y/z) indicate statistical support by MrBayes, PhyML and RAxML (posterior probability, bootstrap and bootstrap, respectively). Values for highly supported nodes have been replaced by symbols, as indicated. Species names are colour-coded based on their bioenergetic mode. Full details and accession numbers for all protein sequences used are given in Table S1. The tree is rooted at the N-ATPase clade, previously reported to be the result of horizontal gene transfer in a variety of species, all of which also contain a canonical ATPF_0_F_1_ (apart from the two *Methanosarcina* species shown which also have a canonical ATPV). The tree confidently separates the major bacterial taxonomic lineages, but with limited support for their branching order: reasonable support is only provided for one subgroup containing the chlorobi and the bacteroidetes. Two species-specific duplications (in *Photobacterium profundum* and *Pelobacter carbinolicus*) are highlighted with a red “>”. Two further duplications within the tenericutes are highlighted with a red “-” after the species name; this duplication likely happened before the split between *Mycoplasma agalactiae* and *Ureaplasma parvum*.
(EPS)

Figure S4 **Phylogenetic reconstruction of ATPF1D.** The tree shown is the best Bayesian topology, based on 189 sequences and 180 amino acid positions (length after trimming; median sequence length before trimming: 181). Numerical values at the nodes of the tree (x/y/z) indicate statistical support by MrBayes, PhyML and RAxML (posterior probability, bootstrap and bootstrap, respectively). Values for highly supported nodes have been replaced by symbols, as indicated. Species names are colour-coded based on their bioenergetic mode. Full details and accession numbers for all protein sequences used are given in Table S1. The tree is rooted at *Thermotogae*, which is generally accepted as being one of the ancestral lineages of the bacteria (N-ATPase has no ATPF1D). The tree confidently separates the major bacterial taxonomic lineages, but with no clear support for their branching order. One species-specific duplication is highlighted with a red “>” in *Pelobacter carbinolicus*. Two further duplications are highlighted with a red “-” after the species name: in *Photobacterium profundum* the (lineage-specific) duplication either occurred before the split from other closely-related species or represents HGT from other gamma-proteobacteria; the evolutionary history of the duplication in *Ureaplasma parvum* cannot be clearly inferred from the phylogenetic analysis; it appears to be species-specific in the PhyML and RaxML trees, but is not statistically supported by high bootstrap values.
(EPS)

Figure S5 **Phylogenetic reconstruction of ATPF1E.** The tree shown is the best Bayesian topology, based on 221 sequences and 138 amino acid positions (length after trimming; median sequence length before trimming: 137). Numerical values at the nodes of the tree (x/y/z) indicate statistical support by MrBayes, PhyML and RAxML (posterior probability, bootstrap and bootstrap, respectively). Values for highly supported nodes have been replaced by symbols, as indicated. Species names are colour-coded based on their bioenergetic mode. Full details and accession numbers for all protein sequences used are given in Table S1. The tree is rooted at the N-ATPase clade, previously reported to be the result of horizontal gene transfer in a variety of species, all of which also contain a canonical ATPF_0_F_1_ (apart from the two *Methanosarcina* species shown which also have a canonical ATPV). The tree confidently separates the major bacterial taxonomic lineages, but with no clear support for their branching order. Three species-specific duplications are highlighted with a red “>” in *Pelobacter carbinolicus*, *Photobacterium profundum*, and *Mariprofundus ferrooxydans*. Lineage-specific duplications (the duplication either occurred before the split from other closely-related species or represents HGT from other closely-related species) are highlighted with a red “-” after the species name: one duplication seems to have occurred before the split between *Desulfovibrio magneticus* and *Desulfovibrio* sp. FW1012B in the delta-proteobacteria; another duplication is seen in the beta-proteobacterium *Thiobacillus denitrificans* (which may represent HGT from other gamma-proteobacteria); finally a duplication occurred before the split between the gamma-proteobacteria *Acidithiobacillus ferrooxidans*, *Acidithiobacillus ferrivorans* and *Acidithiobacillus caldus*, with a further species-specific duplication in *Acidithiobacillus ferrooxidans*.
(EPS)

Figure S6 **Phylogenetic reconstruction of ATPF1G.** The tree shown is the best Bayesian topology, based on 215 sequences and 291 amino acid positions (length after trimming; median sequence length before trimming: 291). Numerical values at the nodes of the tree (x/y/z) indicate statistical support by MrBayes, PhyML and RAxML (posterior probability, bootstrap and bootstrap, respectively). Values for highly supported nodes have been replaced by symbols, as indicated. Species names are colour-coded based on their bioenergetic mode. Full details and accession numbers for all protein sequences used are given in Table S1. The tree is rooted at the N-ATPase clade, previously reported to be the result of horizontal gene transfer in a variety of species, all of which also contain a canonical ATPF_0_F_1_ (apart from the two *Methanosarcina* species shown which also have a canonical ATPV). The tree confidently separates the major bacterial taxonomic lineages, but with limited support for their branching order: reasonable support is only provided for one subgroup containing the chloroflexi, beta-gamma-proteobacteria and the verrucomicrobia, and another subgroup containing the actinobacteria and the planctomycetes (as well as the spirochaete *Leptospira interrogans* and the gemmatimonadete *Gemmatimonas aurantiaca*). One species-specific duplication in *Pelobacter carbinolicus* is highlighted with a red “>”. Two further duplications are highlighted with a red “-” after the species name; in *Photobacterium profundum* the duplication either occurred before the split from other closely-related species or represents HGT from other gamma-proteobacteria; the duplication in *Aquifex aeolicus* represents a very divergent sequence which groups with the dictyoglomi in the MrBayes and PhyML trees.
(EPS)

Figure S7 **Phylogenetic reconstruction of ATPI (K02116).** The analysis is based on 275 sequences and 98 amino acid positions (length after trimming; median sequence length before trimming: 114), including “sI” proteins containing the pfam03899-ATP_synthI domain, “I” proteins containing the pfam09527-ATPase_gene1, and “R” proteins containing the pfam12966-atpR domain (found in the N-ATPase locus). Species names are colour-coded based on their bioenergetic mode. Full details and accession numbers for all protein sequences used are given in Table S1. The tree shown is based on PhyML analysis, as phylogenetic analysis with the same set of sequences using MrBayes failed to converge on a tree, and the RaxML tree had very bad resolution. In contrast to the trees for the other subunits, the bootstrap support for most branches is not significant; the low resolution and low bootstrap support are probably due to the short sequence length and high divergence of the ATPI sequences. Nevertheless, the tree does separate a cluster of the “I” proteins (which contain the pfam09527-ATPase_gene1 domain) to the left of the dotted grey line, and another cluster containing the “sI” proteins (pfam03899-ATP_synthI domain) and “R” proteins (pfam12966-atpR domain) to the right of the grey dotted line. Based on the gene locus organization and the protein sizes, the genes encoding the “sI” and “R” proteins look like duplications of the “I” gene, and the PhyML tree indeed supports this hypothesis.
(EPS)

Table S1 **Details of the species used in this study, including species name abbreviations, and accession numbers of the sequences used for the phylogenetic analyses.**
(XLS)

Dataset S1 **Masked alignments are provided for all the sequence datasets used to construct the trees presented in the manuscript.** Each file is for one dataset and is named based on the corresponding ATPF_0_F_1_ subunit. The files are in nexus format, which can be viewed either as simple text, or with a variety of programs that support nexus files (e.g. McClade). The last row of each alignment, entitled “mask” has an “I” for each position which was included in the final trimmed alignment, used in the phylogenetic analysis. Gaps in the alignment which are not marked with “I” were manually removed in McClade.
(ZIP)
